# A phase 1/2 study of gilteritinib in combination with chemotherapy in newly diagnosed patients with AML in Asia

**DOI:** 10.1007/s12185-024-03840-x

**Published:** 2024-11-06

**Authors:** Masashi Sawa, Toshihiro Miyamoto, Hee-Je Kim, Yasushi Hiramatsu, June-Won Cheong, Takayuki Ikezoe, Tomoki Naoe, Koichi Akashi, Satoshi Morita, Masanori Kosako, Moyu Ikegaya, Wataru Terada, Takeshi Kadokura, Jason Hill, Shuichi Miyawaki, Stanley C. Gill, Alexandra Heinloth, Nahla Hasabou

**Affiliations:** 1 Department of Hematology and Oncology, Aichi, Japan; 2 Department of Hematology, Kanazawa University, Ishikawa, Japan; 3 Catholic Hematology Hospital, Seoul St. Mary’s Hospital, College of Medicine, The Catholic University of Korea, Seoul, South Korea; 4 Department of Hematology and Oncology, Japanese Red Cross Society Himeji Hospital, Hyogo, Japan; 5 Department of Internal Medicine, Severance Hospital, Yonsei University Health System, Seoul, South Korea; 6 Department of Hematology, Fukushima Medical University Hospital, Fukushima, Japan; 7 National Hospital Organization Nagoya Medical Center, Aichi, Japan; 8 Department of Medicine and Biosystemic Science, Kyushu University Hospital, Fukuoka, Japan; 9 Kyoto University Graduate School of Medicine, Kyoto, Japan; 10 Astellas Pharma Inc, Tokyo, Japan; 11 Astellas Pharma US, Inc., Northbrook, IL USA; 12 Division of Hematology, Tokyo Metropolitan Otsuka Hospital, Tokyo, Japan

**Keywords:** Acute myeloid leukemia, Chemotherapy, Gilteritinib, Newly diagnosed, *FLT3* mutation

## Abstract

**Objective:**

This interim analysis of a phase 1/2, open-label, single-arm study assessed the safety, efficacy, and pharmacokinetics of gilteritinib plus chemotherapy in adults with newly diagnosed FLT3 mutation-positive acute myeloid leukemia.

**Methods:**

In sequential phase 1 and 2 studies, induction and consolidation therapy with gilteritinib 120 mg/day plus chemotherapy (induction: idarubicin/cytarabine once daily; consolidation: cytarabine twice daily) was followed by maintenance gilteritinib 120 mg/day monotherapy. Endpoints included maximum tolerated dose (MTD), recommended expansion dose (RED), and dose-limiting toxicity (phase 1), and complete remission (CR) rate following induction therapy (primary endpoint), overall survival (OS), safety, and pharmacokinetics (phase 2).

**Results:**

In phase 1, MTD was not reached and RED was 120 mg/day. In phase 2, the CR rate was 50.0% after induction (90% confidence interval [CI] 40.4, 59.6); however, the lower confidence limit did not exceed the pre-defined 55% benchmark. Composite CR (CRc) rates were high following induction (86.6%, 95% CI [77.3, 93.1]), consolidation, and maintenance therapy (87.8%, 95% CI [78.7, 94.0], each). The probability of OS was 86.6% at 12 months. No new safety findings were reported.

**Conclusion:**

In this interim analysis, gilteritinib 120 mg/day in combination with chemotherapy was well tolerated, with similar CRc rates to previous studies.

**Supplementary Information:**

The online version contains supplementary material available at 10.1007/s12185-024-03840-x.

## Introduction

Acute myeloid leukemia (AML) is the most common form of myeloid leukemia worldwide [1], with increasing incidence associated with advancing patient age [2]. Furthermore, AML is associated with high rates of mortality and morbidity [3–5], particularly in patients with mutations in the *FMS-like tyrosine kinase 3* gene (*FLT3*^*mut+*^) [6].

Intensive chemotherapy regimens are the standard treatment for newly diagnosed (ND) and relapsed/refractory (R/R) AML, which may be followed by hematopoietic stem cell transplantation (HSCT) in cases of primary refractory disease as recommended by European LeukemiaNet guidelines [7]. However, not all patients with AML are eligible for standard treatment (for instance, due to advanced age [7–9]), and 50–70% of patients who achieve complete remission subsequently relapse [10]. Compared with the general AML population, patients with *FLT3* internal tandem duplication (ITD) mutations have a higher risk of relapse and a shorter overall survival, while the impact of *FLT3* tyrosine kinase domain (TKD) mutations on outcomes is less clear [11]. As such, there is an unmet need for alternative therapies in patients with ND AML and ND *FLT3*^*mut+*^ AML.

Gilteritinib is a selective, oral FLT3 inhibitor with activity against both ITD and TKD mutations, which offers an alternative to standard chemotherapy [12]. The safety and efficacy of gilteritinib monotherapy versus salvage chemotherapy in patients with R/R *FLT3*^*mut+*^ AML have been demonstrated in the phase 3 ADMIRAL study [13]. Subsequently, gilteritinib received approval from the European Medicines Agency [14] and U.S. Food and Drug Administration [15], as well as the Ministry of Health Labor and Wealth in Japan for the treatment of patients with R/R *FLT3*^*mut+*^ AML [16].

Alongside the approval of gilteritinib in patients with R/R AML, studies have sought to understand how patients with *FLT3*^*mut+*^ AML might benefit from a combination of gilteritinib with chemotherapy [17, 18]. A pre-clinical study in xenograft mouse models found that gilteritinib in combination with chemotherapy might have synergistic cytotoxic effects and offer improved outcomes versus each respective monotherapy in the *FLT3*^*mut+*^ AML setting [17]. Further to this, a phase 1 study in the United States demonstrated that gilteritinib plus chemotherapy was well tolerated and resulted in favorable antileukemic responses in patients with ND *FLT3*^*mut+*^ AML [18].

Here we present interim results from a phase 1/2 study investigating the optimal dose, safety, and efficacy of gilteritinib in combination with cytarabine/idarubicin chemotherapy in Asian patients with ND AML.

## Materials and methods

### Ethics

This study was conducted in accordance with Good Clinical Practice and consensus ethical principles derived from international guidelines including the Declaration of Helsinki, Council for International Organizations of Medical Sciences international ethical guidelines**,** and applicable International Council for Harmonisation of Technical Requirements for Pharmaceuticals for Human Use Guidelines for Good Clinical Practice. Prior to study commencement, the protocol and any amendments were reviewed and approved by an Institutional Review Board or Independent Ethics Committee at each study site. Written informed consent was provided by each patient prior to initiation of any study**-**related procedures.

### Study design

**Fig. 1:**
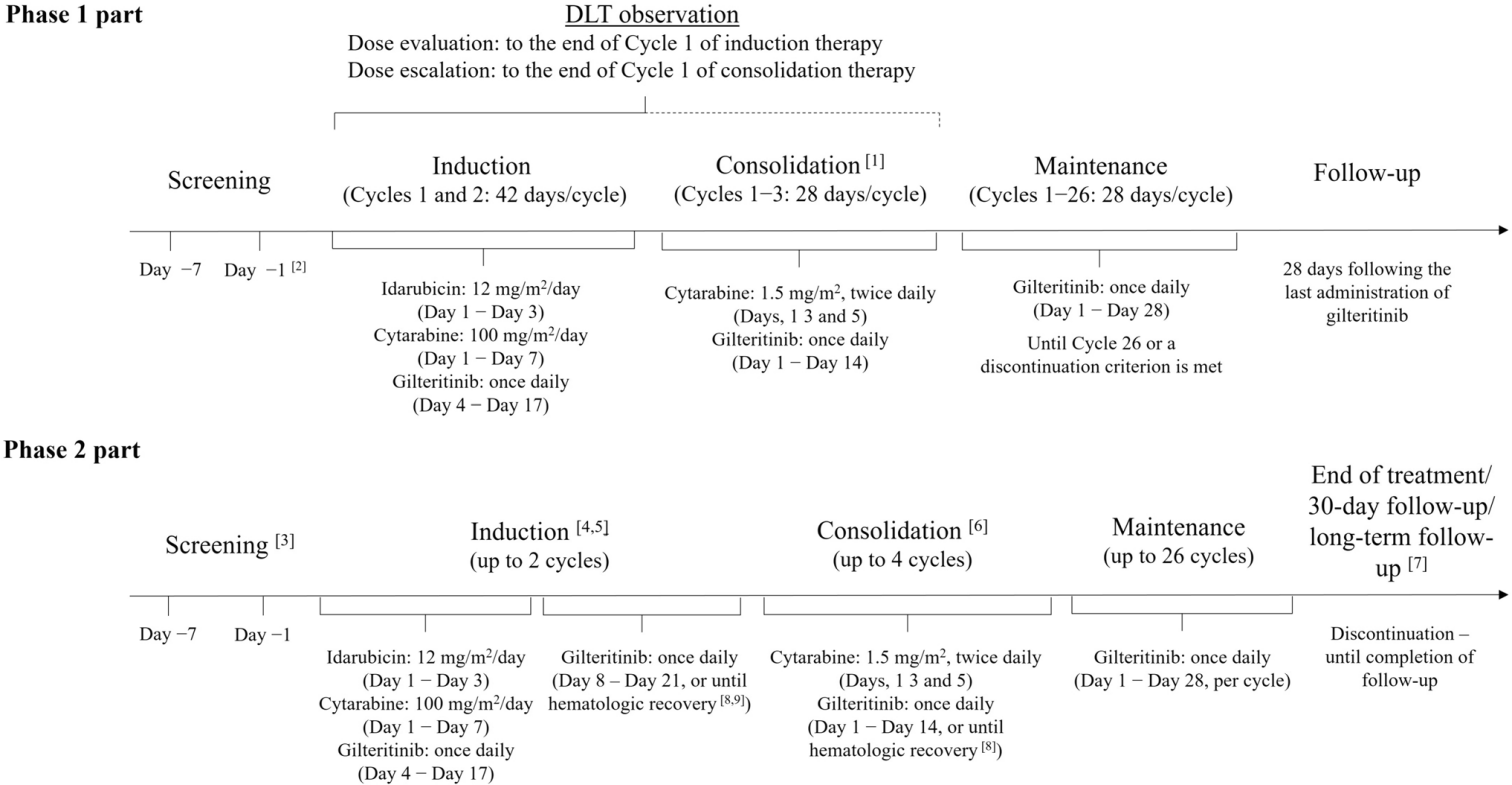
Study flow diagram [1] Cycle 1 was to be started without delay when a patient had reached remission with blood count recovery (within 57 days from the start of the last cycle of induction therapy). Subsequent cycles were to be started after completion of the assessment on Day 28 of the previous cycle; subsequent cycles were to be started after Day 22 of the previous cycle, following recovery of the patient’s blood count. [2] Treatment was allowed to be started on the day informed consent was obtained when the investigator or subinvestigator judged that immediate treatment was required due to rapid proliferative disease progression. In this case, among the test items specified in both screening and Day 1 of Cycle 1, tests to determine body height, body weight, vital signs, and ECOG PS and laboratory tests were not required to be performed twice. [3] Consent, screening, and Cycle 1 Day 1 of the induction period were allowed to be performed on the same day if investigator(s) judged that it would be necessary due to rapid disease progression and patient met all inclusion/exclusion criteria for pre-registration, except the following inclusion criteria: FLT3-ITD and/or TKD mutation positive, AST/ALT <3 x ULN, serum magnesium ≥ institutional LLN. Cycle 1 Day 1 had to be initiated within 7 days of consent. [4] Number of days in 1 cycle was not defined in the phase 2 part. Each cycle was allowed to be extended until blood recovery was observed. Timing to initiate the second cycle or next treatment period was determined by investigators based on patient condition. [5] Patients had to fulfill all inclusion and exclusion criteria by the end of Day 7 in order to complete registration on Day 8. [6] Patients who were eligible for HSCT were allowed to receive HSCT without consolidation therapy. Patients who received HSCT were allowed to return to the study if the following conditions were met: patient was 30–90 days post-HSCT, patient had successful engraftment as demonstrated by ANC ≥500/mm3 and platelets ≥20000/mm3 without transfusions; patient did not have ≥Grade 2 acute GVHD; and patient was in CRc. [7] Follow-up period was 3 years from treatment initiation for the last enrolled patient or completion of 30-day follow-up of the last patient(s), whichever was longer. [8] Preferably, the following cycle was started after full hematologic recovery (defined as neutrophil count ≥1000/mm3, platelet count ≥100,000/mm3), allowing formal assessment of response. [9] Patients who achieved full hematologic recovery prior to Day 42 were preferred to perform the Day 42 scheduled visit for patient safety. If the patient achieved more than a partial remission, the patient was allowed to continue to the next therapy or proceed to HSCT. If refractory disease was confirmed, scheduled visits were allowed to be skipped to perform Cycle 2 of induction therapy. ALT; alanine aminotransferase; ANC, absolute neutrophil count; AST, aspartate aminotransferase; DLT, dose limiting toxicity; ECOG, Eastern Cooperative Oncology Group; HSCT, hematopoietic stem cell transplantation; ITD, internal tandem duplication; LLN, lower limit of normal; PS, performance status; TKD, tyrosine kinase domain; ULN, upper limit of normal

This was a phase 1/2, open-label, single-arm study conducted in 52 centers across Japan, Korea, and Taiwan between 26 February 2015 and 25 August 2021 (interim data analysis cut-off date). The study comprised sequential phase 1 and phase 2 parts. Treatment administration for both phase 1 and phase 2 is detailed in Fig. 1.

Phase 1 of this study was split into two parts. First, the dose-evaluation part assessed the maximum tolerated dose (MTD) of gilteritinib based on the occurrence of dose-limiting toxicities (DLTs). Second, the dose-escalation part confirmed the safety of the identified tolerated dose of gilteritinib. The starting dose of gilteritinib in cycle 1 of the induction period was 120 mg/day; the next dose level (120 mg/day or 80 mg/day) was determined sequentially using Bayesian continual reassessment and was informed by discussion between the investigator, medical advisor, and the sponsor.

In the dose-evaluation part, DLTs were assessed until the end of cycle 1 of the induction period; in the dose-escalation part, DLTs were assessed until the end of cycle 1 of the consolidation period. DLTs were categorized as being possibly, probably, or definitely related to induction or consolidation therapies including the study drug (Supplementary Methods).

The phase 2 part of the study was initiated only after the achievement of the phase 1 objectives. The phase 2 treatment pathway was similar to that in the phase 1 part (Fig. 1) but included the following differences: patients with an identified donor and successful response were permitted to undergo HSCT (per each institution’s assessment) and continued to receive maintenance therapy without leaving the study; and patients could resume treatment with the study drug after HSCT initiation if specified criteria were met (Supplementary Methods). Additionally, long-term follow-up is being conducted over a period of 3 years from treatment initiation for the last enrolled patient or completion of 30-day follow-up of the last patient(s), whichever is longer. All patients enrolled in the phase 2 part received gilteritinib at the recommended dose established in the phase 1 part.

The study is ongoing and is registered at ClinicalTrials.gov (NCT02310321). This paper presents an interim analysis, for which the data cut-off corresponded to the date of the last enrolled patient completing the phase 2 induction period.

### Patients

Eligible patients were aged ≥18 and <70 years old (phase 1 part) or considered adult according to local regulations at the time of consent (phase 2 part). Patients had to have ND AML according to World Health Organization 2008 (phase 1) or 2017 (phase 2) criteria, documented within 28 days prior to enrollment. Patients had an Eastern Cooperative Oncology Group (ECOG) performance status (PS) of ≤2; patients with an ECOG PS of 2 were eligible for the phase 2 study part only if investigators suspected that their primary disease-related symptoms were the causes of their PS score. Patients were required to meet protocol-specified clinical laboratory test criteria (see Supplementary Methods). Patients enrolled in the phase 2 part of the study were also positive for *FLT3*-ITD or *FLT3*-TKD mutations, as determined by the central laboratory from bone marrow or whole blood taken at the screening visit, using a PCR-based assay (Leukostrat CDx *FLT3* Mutation Assay) [19]. Pre-registration for the phase 2 part was conducted when a patient met all eligibility criteria except for *FLT3*-ITD and/or *FLT3*-TKD mutation positivity; serum aspartate aminotransferase (AST) and alanine aminotransferase (ALT) < 3 × upper limit of normal (ULN); and serum magnesium ≥ institutional lower limit of normal.

Patients with acute promyelocytic leukemia, breakpoint cluster region–Abelson oncogene-positive leukemia (chronic myelogenous leukemia in blast crisis), active malignant tumors other than AML or myelodysplastic syndrome, or clinically active central nervous system leukemia, were excluded.

### Outcomes

In the phase 1 part of the study, the primary objectives were to determine the maximum tolerated dose (MTD; defined in Supplementary Methods) and/or recommended expansion dose (RED; defined in Supplementary Methods) of gilteritinib in combination with induction chemotherapy, based on the onset of DLT. In addition, the safety and tolerability of gilteritinib as part of combination induction and consolidation therapy, and as subsequent maintenance therapy were evaluated.

In the phase 2 part of the study, the primary efficacy outcome was the rate of complete remission (CR) following induction therapy. Secondary outcomes included overall survival (OS); event-free survival (EFS); relapse-free survival (RFS); rates of CR, CR without minimal residual disease (MRD), CR with partial hematological recovery (CRh), CR/CRh, and composite CR (CRc, composed of the sum of CR, CRp and CR with incomplete hematologic recovery [CRi]); durations of CR, CRh, CR/CRh, and CRc; and pharmacokinetic (PK) parameters. An *ad hoc* additional analysis was conducted by aligning the definition for CRi with the QuANTUM-First study of quizartinib [20] (Supplementary Methods). Response rates are presented using the best overall response: patients who achieved CR in induction therapy were counted as CR even if not reported with CR in consolidation and maintenance therapy. Exploratory outcomes included HSCT rate; cumulative incidence of relapse or death after the first CR; time to hematologic recovery after each treatment cycle; MRD; and MRD-negative CR rate after induction therapy and for the overall treatment period. MRD negativity was defined as a summed *FLT3*-ITD signal ratio ≤10^–4^ for any post-baseline sample. Full definitions of efficacy outcomes, including the determination of hematologic recovery, are given in the Supplementary Methods.

Efficacy assessments were based on bone marrow aspirates and/or biopsies at screening and at any time on or after day 28 in Cycle 1 of induction therapy, prior to initiation of consolidation/maintenance therapy, and thereafter during maintenance therapy according to institutional guidelines. In both phase 1 and 2 parts of the study, safety outcomes were evaluated in terms of adverse events (AEs), laboratory assessments, vital signs, and electrocardiogram (ECG) measurements. All AEs were coded using the Medical Dictionary for Regulatory Activities (MedDRA v23.0) and graded using the National Cancer Institute – Common Terminology Criteria v4.0 for Adverse Events (NCI-CTCAE) [21]. A treatment-emergent AE (TEAE) was defined as an AE observed after initiation of gilteritinib and within 28 (phase 1) or 30 days (phase 2) following the last administration of gilteritinib. All TEAEs were summarized by system organ class and preferred term.

QT interval prolongation was defined based on actual values and NCI-CTCAE grading [21]. Change in corrected QT interval from baseline was also evaluated, although these changes are not attributable to an NCI-CTCAE grade.

### Statistical analyses

In the phase 1 part, a sample size of three DLT-evaluable patients per dose level was considered adequate to assess gilteritinib MTD and RED; in the dose-escalation part, the target sample size was three DLT-evaluable patients at the recommended dose established in the dose-evaluation part. Dose evaluation/escalation analyses were conducted in the dose-determining analysis set, comprising all patients who received ≥80% of the assigned dose of gilteritinib during the DLT assessment period and were able to be assessed adequately for safety. Patients considered unevaluable for DLT were replaced by another patient in the cohort. Frequency and posterior mean of the DLT incidence were calculated for each dose across both dose evaluation/escalation parts.

In the phase 2 part, the target sample size was 80 patients; an evaluable sample size of 70 patients was estimated to provide >80% power to detect a 15% increase in CR rate from 55% (benchmark based on the placebo arm of the RATIFY study [22]) to 70% at a one-sided significance level of 0.05. Primary efficacy analyses were conducted in the full analysis set (FAS), comprising all patients who received ≥1 dose of the study drug and had ≥1 post-baseline bone marrow assessment. Pharmacokinetic analyses were conducted in the pharmacokinetic analysis set, comprising all patients who received ≥1 dose of the study drug, and had available drug concentration data for at least one-time point after drug initiation. MRD was assessed in the MRD analysis set (MAS), comprising all patients who received ≥1 dose of the study drug, were confirmed as *FLT3-*ITD positive, and had both baseline and post-baseline MRD data.

For the rate of CR following induction therapy (primary efficacy outcome), the 2-sided 90% exact confidence interval (CI) was calculated by the Clopper-Pearson method; the lower limit of the CI was compared to a benchmark of 55%, based on the CR rate in the placebo arm of the RATIFY study [22]. For secondary efficacy outcomes, survival rates (OS, EFS, and RFS) and duration of remission (CR and CRc) were summarized by the number of events and described using the Kaplan-Meier method for time-to-event outcomes. Binary outcomes for rates of remission (rates of CR, CRc, CRp, and CRi and CR/CRh) were described using a 2-sided 95% exact CI. Plasma gilteritinib concentration data were used to calculate pharmacokinetic parameters and summarized descriptively. An additional *ad hoc* analysis was conducted to determine median follow-up for OS, with two-sided 95% CI calculated using the reverse Kaplan–Meier method.

Safety analyses in both phase 1 and 2 study parts were conducted in the safety analysis set (SAF), comprising all patients who received ≥1 dose of gilteritinib. TEAEs, laboratory assessments, vital signs and ECG measurements were summarized descriptively.

No statistical imputation was used to handle missing data. All statistical analyses were conducted using the SAS (Version 9.4) software package.

This study is ongoing, and the current analysis presents interim results at data cut-off (25 August 2021), at which time all patients in the phase 2 part have completed induction therapy. A final analysis of study outcomes will be conducted after study completion.

## Results

### Patient disposition, demographics, and baseline characteristics

In the phase 1 part, 16 patients were enrolled, of whom two patients discontinued prior to induction chemotherapy and one patient who received gilteritinib 40 mg was excluded from all analyses (Supplementary Figure S1). The remaining 13 patients received chemotherapy plus gilteritinib, with a median (range) duration of gilteritinib exposure of 28.0 (14−114) days.

In the phase 2 part, 213 patients were pre-registered, of whom 212 received chemotherapy; 84 patients continued to receive gilteritinib 120 mg/day and were included in the SAF (Supplementary Figure S1). Median (range) duration of gilteritinib exposure was 37.5 (3−367) days. Two of these 84 patients did not have a post-baseline bone marrow assessment and were therefore excluded from the FAS.

Most patients only received one cycle of induction therapy in both phase 1 (11/13, 84.6%) and phase 2 (71/84, 84.5%) parts.

Of the 84 patients who received gilteritinib in the phase 2 part, 62 patients proceeded to the consolidation and/or maintenance period(s). At data cutoff (25 August 2021 for this interim analysis), 57 patients had entered the consolidation period; of these, 9 patients discontinued the consolidation period without proceeding to the maintenance period and 48 proceeded on to maintenance therapy. Additionally, four patients skipped the consolidation period and had already entered maintenance at data cut off. In total, 28 patients initiated maintenance therapy; of these, 2 patients discontinued, and 26 patients were still receiving treatment as of the data cutoff date. The numbers of patients included in the analysis populations for each of the phase 1/2 parts are included in Supplementary Table S1.

In the phase 1 part, 8/13 (61.5%) patients were male and the median (min–max) age was 50.0 (26–69) years; 12/13 (92.3%) patients were aged <65 years (Table 1). The median (min–max) body mass index (BMI) was 21.38 (17.9–30.2) kg/m^2^ and a majority of patients (9/13, 69.2%) had an ECOG PS of 0 (Table 1). The median (min–max) duration of AML disease at baseline in phase 1 patients was 0.13 (0.0–0.5) months (Supplementary Table S2); 4/13 (30.8%) patients were also positive for *FLT3*-ITD at baseline.

**Table 1:** Patient baseline characteristics (SAF)

	Phase 1 part			Phase 2 part
	Dose evaluation (*N* = 3)	Dose expansion (*N* = 10)	Total (*N* = 13)	Total (*N* = 84)
Sex, *n* (%)				
Male	1 (33.3)	7 (70.0)	8 (61.5)	41 (48.8)
Female	2 (66.7)	3 (30.0)	5 (38.5)	43 (51.2)
Age (years)				
Mean (SD)	48.3 (14.0)	49.9 (15.0)	49.5 (14.2)	51.0 (14.4)
Median (min–max)	44.0 (37–64)	53.0 (26–69)	50.0 (26–69)	52.0 (20–77)
Age group, *n* (%)				
<65 years	3 (100.0)	9 (90.0)	12 (92.3)	68 (81.0)
≥65 years	0	1 (10.0)	1 (7.7)	16 (19.0)
Region, *n* (%)				
Japan	–	–	–	59 (70.2)
Korea	–	–	–	22 (26.2)
Taiwan	–	–	–	3 (3.6)
Weight, kg				
Mean (SD)	59.87 (26.49)	61.92 (12.56)	61.45 (15.36)	62.42 (13.38)
Median (min–max)	52.00 (38.2–89.4)	59.35 (45.3–86.4)	58.80 (38.2–89.4)	60.10 (40.5–90.6)
Height at screening, cm				
Mean (SD)	158.20 (13.13)	164.34 (8.76)	162.92 (9.67)	163.55 (9.40)
Median (min–max)	156.50 (146.0–172.1)	166.35 (153.7–177.7)	165.00 (146.0–177.7)	163.00 (146.2–189.0)
BMI, kg/m^2^				
Mean (SD)	23.11 (6.34)	22.77 (3.26)	22.85 (3.84)	22.88 (3.56)
Median (min–max)	21.23 (17.9–30.2)	21.43 (18.9–28.2)	21.38 (17.9–30.2)	22.55 (16.2–31.0)
BSA, m^2^				
Mean (SD)	1.59 (0.39)	1.67 (0.20)	1.65 (0.24)	1.66 (0.21)
Median (min–max)	1.50 (1.3–2.0)	1.68 (1.4–2.0)	1.67 (1.3–2.0)	1.66 (1.3–2.2)
ECOG PS, *n* (%)				
0	1 (33.3)	8 (80.0)	9 (69.2)	30 (35.7)
1	2 (66.7)	2 (20.0)	4 (30.8)	48 (57.1)
2	0	0	0	6 (7.1)
3	0	0	0	0
4	0	0	0	0
*FLT3*-ITD mutation status at baseline, *n* (%)				
Negative	0	5 (50.0)	5 (38.5)	15 (17.9)
Positive	2 (66.7)	2 (20.0)	4 (30.8)	69 (82.1)
Unknown	1 (33.3)	3 (30.0)	4 (30.8)	ND
*FLT3* point mutation status at baseline, *n* (%)				
Negative	0	1 (10.0)	1 (7.7)	–
Positive	0	0	0	–
Unknown	3 (100.0)	9 (90.0)	12 (92.3)	–
*FLT3*-TKD mutation status at baseline, *n* (%)				
Negative	–	–	–	64 (76.2)
Positive	–	–	–	20 (23.8)
Unknown	–	–	–	ND
*FLT3* final mutation status at baseline, *n* (%)				
Negative	–	–	–	0
Positive	–	–	–	84 (100.0)
Unknown	–	–	–	ND

*BMI* body mass index, *BSA* body surface area, *ECOG* Eastern Cooperative Oncology Group, PS performance status, *FLT3* FMS-like tyrosine kinase 3, *ITD* internal tandem duplication, *max* maximum, *min* minimum, *ND* not detected, *SAF* safety analysis set, *SD* standard deviation, *TKD* tyrosine kinase domain

In phase 2 of the study, 41/84 (48.8%) patients were male and the median (min–max) age was 52.0 (20–77) years; 68/84 (81.0%) patients were aged <65 years (Table 1). The median (min–max) BMI was 22.55 (16.2–31.0) kg/m^2^ and over half of patients (48/84, 57.1%) had an ECOG PS of 1; 69/84 (82.1%) and 20/84 (23.8%) patients were positive for *FLT3*-ITD and *FLT3*-TKD mutations, respectively (Table 1). The median (min–max) duration of AML disease at baseline in phase 2 patients was 0.16 (0.0–0.5) months (Supplementary Table S2).

### Phase 1: Safety

#### MTD and/or RED

DLTs were reported in 1/13 (7.7%) patients who received gilteritinib 120 mg/day in the dose-expansion study part; the patient experienced grade 3 non-hematologic or extramedullary toxicity (diarrhea), which was considered possibly related to gilteritinib. No patients received gilteritinib 80 mg/day. In the 13 patients who received gilteritinib 120 mg/day, the posterior mean of DLT incidence estimated using Bayesian continual reassessment was 0.103, which was less than the pre-specified threshold of 0.33. MTD was therefore not reached, and the RED was determined to be 120 mg/day.

#### Adverse events

All patients in phase 1 of the study experienced at least one AE and at least one grade 3 or higher TEAE (Table 2, Supplementary Table S3). The most common grade 3 TEAE was febrile neutropenia (12/13, 92.3%) (Supplementary Table S3). Serious TEAEs were reported in 3/13 (23.1%) patients: abnormal liver function test (2/13, 15.4%) and abnormal hepatic function test (1/13, 7.7%). TEAEs leading to withdrawal of treatment occurred in 2/13 (15.4%) patients. No deaths during the study period (from the first gilteritinib administration) through follow-up were reported during the phase 1 part (Table 2).

**Table 2:** Overview of TEAEs and deaths (SAF)

	Phase 1 part (*N* = 13)		Phase 2 part (*N* = 84)		Combined phase 1 & 2 parts (*N* = 97)	
	*n* (%)	No. of events	*n* (%)	No. of events	*n* (%)	No. of events
Any TEAEs	13 (100.0)	492	84 (100.0)	2170	97 (100.0)	2662
Serious TEAEs^a^	3 (23.1)	5	40 (47.6)	86	43 (44.3)	91
TEAEs leading to withdrawal of treatment	2 (15.4)	2	5 (6.0)	5	7 (7.2)	7
TEAEs leading to death	0	0	4 (4.8)	7	4 (4.1)	7
Grade 3 or higher TEAEs	13 (100.0)	208	79 (94.0)	778	92 (94.8)	986
Death^b^	0	–	8 (9.5)	–	8 (8.2)	–

*TEAE* treatment-emergent adverse event, *SAF* safety analysis set

^a^Includes SAEs upgraded by the sponsor, based on the review of the Sponsor's list of Always Serious terms, if any upgrade was done

^b^All reported deaths after the first gilteritinib administration

### Phase 2: Efficacy

#### Treatment response

**Fig. 2:**
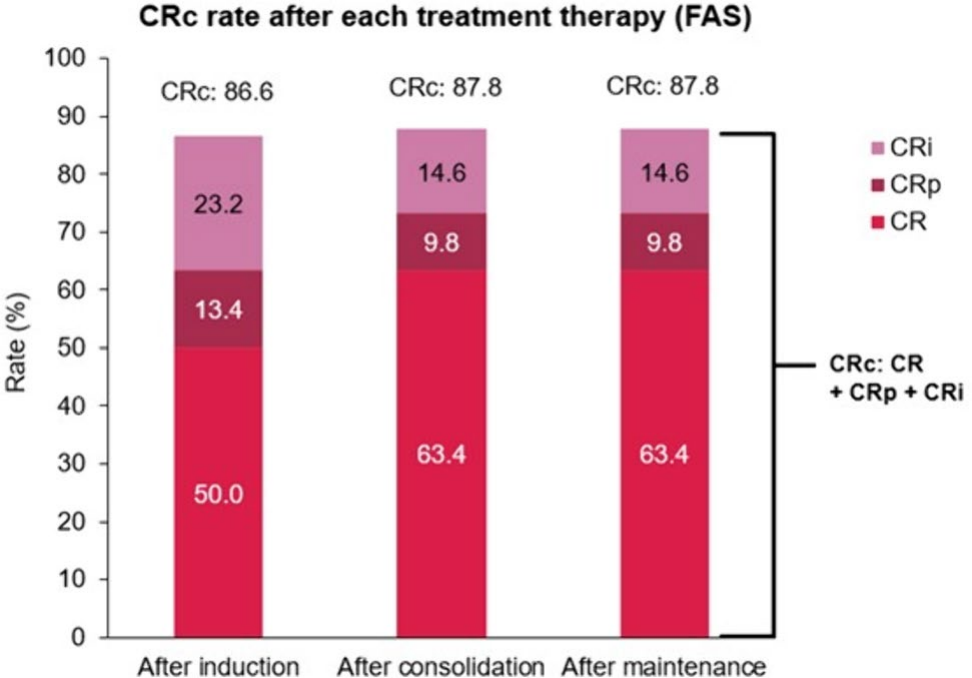
CRc rates after each treatment period (FAS) CR, complete remission; CRc, composite complete remission rate; CRi, complete remission with incomplete hematological recovery; CRp, complete remission with incomplete platelet recovery; FAS, full analysis set

In phase 2 of the study, the CR (90% CI) rate after induction therapy was 50.0% (40.4, 59.6); however, the lower limit of the 90% CI for CR did not exceed the pre-defined benchmark of 55%. The best overall response for CR rate after induction therapy was 50.0% (95% CI 38.7, 61.3), increasing to 63.4% (95% CI 52.0, 73.8) after consolidation and maintenance therapy (data not matured; Table 3, Fig. 2). In contrast, CRc (95% CI) rates were high and stable after induction therapy (86.6% [77.3, 93.1]), and the consolidation and maintenance periods (87.8% [78.7, 94.0], each). This was due to increasing proportions of patients transitioning from CRp/CRi to CR from induction to consolidation therapy (Table 3; Fig. 2).

**Table 3:** Derived response after induction, consolidation and maintenance therapy (phase 2 study; FAS)

BOR, *n* (%) (95% CI)^a^	Phase 2 patients (*N* = 82)		
	After induction therapy	After consolidation therapy	After maintenance therapy
CR	41 (50.0) (38.7, 61.3)	52 (63.4) (52.0, 73.8)	52 (63.4) (52.0, 73.8)
CRp	11 (13.4) (6.9, 22.7)	8 (9.8) (4.3, 18.3)	8 (9.8) (4.3, 18.3)
CRi	19 (23.2) (14.6, 33.8)	12 (14.6) (7.8, 24.2)	12 (14.6) (7.8, 24.2)
CRc	71 (86.6) (77.3, 93.1)	72 (87.8) (78.7, 94.0)	72 (87.8) (78.7, 94.0)
Response	75 (91.5) (83.2, 96.5)	76 (92.7) (84.8, 97.3)	76 (92.7) (84.8, 97.3)
CR/CRh	55 (67.1) (55.8, 77.1)	60 (73.2) (62.2, 82.4)	60 (73.2) (62.2, 82.4)
CR without MRD	15 (18.3) (10.6, 28.4)	27 (32.9) (22.9, 44.2)	27 (32.9) (22.9, 44.2)
CRc without MRD	19 (23.2) (14.6, 33.8)	32 (39.0) (28.4, 50.4)	32 (39.0) (28.4, 50.4)

*BOR* best overall response (patients who achieved CR in induction therapy were counted as CR even if not reported with CR in consolidation and maintenance therapy), *CI* confidence interval, *CR* complete remission, *CRc* composite complete remission (CR + CRp + CRi), *CRi* complete remission with incomplete hematological recovery, *CRp* complete remission with incomplete platelet recovery, *FAS* full analysis set, *HSCT* hematopoietic stem cell transplantation, *MRD* minimal residual disease

For patients who underwent HSCT, any response assessment data after HSCT were not included in this table

^a^95% exact CI was estimated using the binomial distribution

CRc (95% CI) rates without MRD after induction therapy were 23.2% (14.6, 33.8) and 39.0% (28.4, 50.4) after the consolidation and maintenance periods (Table 3). CR/CRh (95% CI) rates were 67.1% (55.8, 77.1) after induction therapy and 73.2% (62.2, 82.4) after the consolidation and maintenance periods (Table 3). The proportion of patients with CRp or CRi decreased from induction to consolidation and maintenance therapy (Fig. 2). In an *ad hoc* analysis, in which the definition of CRi was changed to include a requirement for platelet recovery (≥100,000/mm^3^) and the time period for evaluating the response was restricted to 60 days following day 1 of the last induction cycle, the proportion of patients with CRc was 69.5% (57/82) (Supplementary Table S4). Overall response (95% CI) rates were 91.5% (83.2, 96.5) after induction therapy and 92.7% (84.8, 97.3) after consolidation and maintenance periods (Table 3); duration of response will be calculated at the final analysis.

#### Survival

**Fig. 3:**
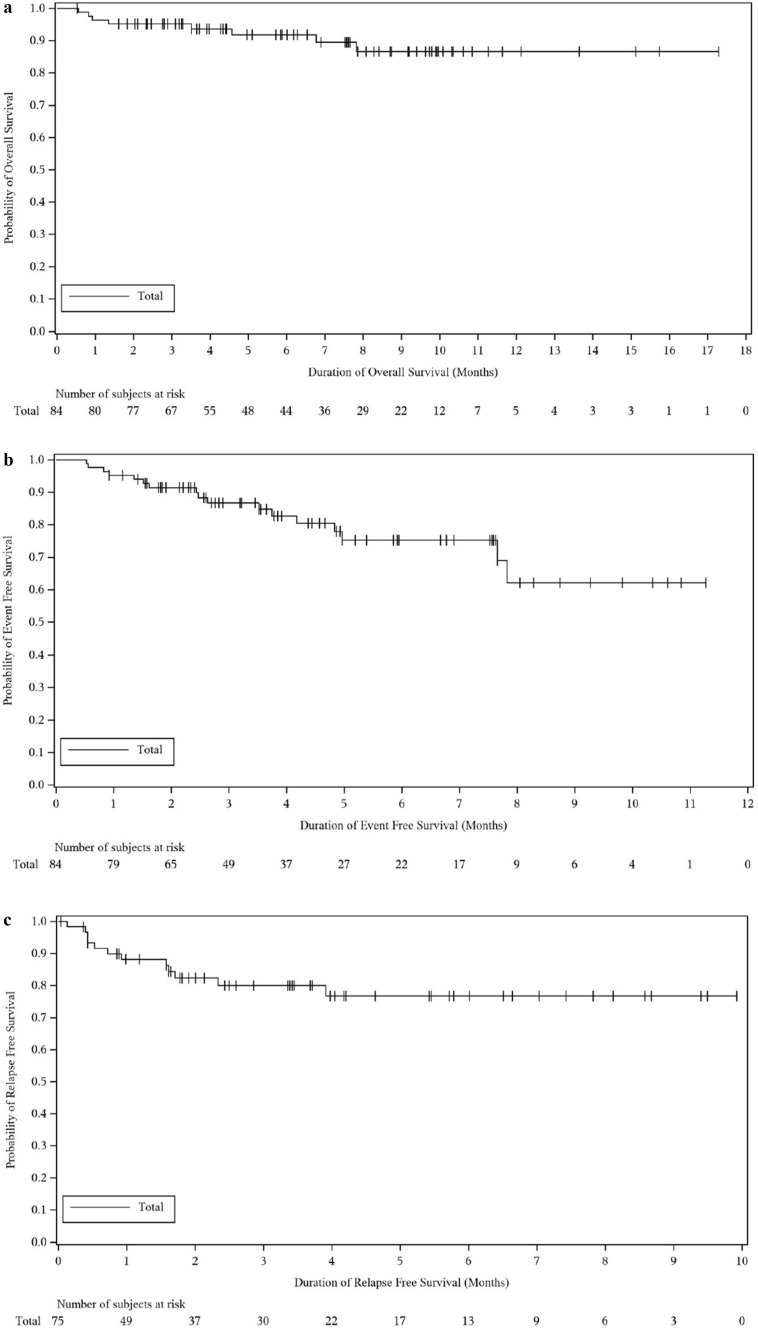
Kaplan-Meier plot of OS (a), EFS (b) and RFS (c) (TTE-FAS) EFS, event-free survival; OS, overall survival; RFS, relapse-free survival; TTE-FAS, time-to-event full analysis set

The probability of OS (95% CI) was 91.8% (82.4, 96.3) at 6 months and 86.6% (73.9, 93.4) at 12 months. A total of 8/84 (9.5%) deaths (OS events) occurred by data cut-off date (Supplementary Table S5), of which 4/8 (50.0%) occurred by Day 60 and 4/8 (50.0%) after treatment discontinuation, respectively. Additionally, 17/84 (20.2%) EFS events (relapse, treatment failure, or death) were reported (Supplementary Table S5); the duration of EFS ranged from 0.5 to 11.3 months. Of 75 patients with the best response of CRc (i.e., patients with RFS), 12 (16.0%) RFS events (relapse or death) were reported (Supplementary Table S5); duration of RFS ranged from <0.1 to 9.9 months. Median OS, EFS and RFS were not reached by the data cut-off date (Fig. 3). In our *ad hoc* analysis, median (95% CI) follow-up for OS was 6.9 (5.1, 8.1) months at data cut-off.

#### MRD, HSCT rate and hematologic recovery

MRD negativity was achieved in 33/61 (54.1%) patients in the MAS; the proportion of patients with MRD negativity was numerically higher following the consolidation period (24/30, 80.0%) than the induction period (22/58, 37.9%). From the start of the study to the data cut-off date, 30/82 (36.6%) patients underwent HSCT. Median time to hematologic recovery was 48.0 (95% CI 46.0, 57.0) days after the start date for Cycle 1 (45/82 events).

### Phase 2: Safety

#### Adverse events

All patients participating in phase 2 of the study experienced at least one AE (Table 2). A total of 79/84 (94.0%) patients experienced grade ≥3 TEAEs, of which the most frequently reported were febrile neutropenia (54/84, 64.3%) platelet count decreased (27/84, 32.1%), and neutrophil count decreased (23/84, 27.4) (Supplementary Table S3). Serious AEs were reported in 40/84 (47.6%) patients (Table 2), of which the most common were sepsis (7/84, 8.3%), and febrile neutropenia, hepatic function abnormal, and pneumonia (5/84, 6.0% each). TEAEs leading to withdrawal of treatment were reported in 5/84 (6.0%) patients, and TEAEs leading to death occurred in 4/84 (4.8%) patients during the entire study period (Table 2). Four of these deaths (4.8%) occurred during the first 60 days of treatment and were attributed to: septic shock in one patient; pneumonia and sepsis in one patient; pneumonia alone in one patient; and brain stem infarction, meningitis, and sinusitis in one patient. One case of pneumonia was considered by the study Investigator to be possibly related to gilteritinib.

### Phase 1 and Phase 2: laboratory assessments, vital signs, and ECG measurements

Elevations of ALT or AST (>5 × ULN) were reported in 23.1 and 11.9% of patients in the phase 1 and phase 2 parts, respectively (Supplementary Table S6). There were no reports of potentially clinically significant vital signs in the phase 1 part. In the phase 2 part of the study, one patient (1/84, 1.2%) reported potentially clinically significant elevations in both systolic and diastolic blood pressure and four (4/84, 4.8%) patients reported potentially clinically significant elevations in pulse rate (Supplementary Table S7).

Prolonged QT interval was reported as a TEAE in 3/13 (23.1%) patients in the phase 1 part and 3/84 (3.6%) patients in the phase 2 part; these TEAEs were determined to be related to gilteritinib for all three patients in the phase 1 part and one patient in the phase 2 part. One (1/8, 12.5%) patient in the phase 1 part and eight (8/84, 9.5%) patients in the phase 2 part experienced a maximum post-baseline change of >60 msec in QT interval corrected for heart rate using Fridericia’s factor (Supplementary Table S8).

### Phase 2: PK

During the induction period, the mean trough concentration of gilteritinib was 522 ng/mL on Cycle 1 Day 15 and 647 ng/mL on Cycle 1 Day 21. Interpatient variability was high (CV >60%) in Cycle 1 regardless of timing, and across both induction and consolidation periods (Supplementary Table S9).

## Discussion

In this interim analysis of a phase 1/2 study of gilteritinib plus chemotherapy in ND patients in Asia with *FLT3*^*mut+*^AML, the MTD for gilteritinib was not reached and the identified RED (120 mg/day) resulted in a CR rate of 50.0% (90% CI 40.4, 59.6) after induction chemotherapy and gilteritinib. The study did not achieve its primary objective since the lower CI limit of 90% for CR did not exceed the pre-defined benchmark of 55%. Despite this, CRc rates were high after the induction period (86.6%), with increasing contribution from CR through consolidation and maintenance therapy. Additionally, CRc rates after induction therapy were comparable in phase 1 and 2 parts and similar to those presented in a preceding phase 1B study conducted in the US [18].

Although findings from the phase 2 part of this analysis report that the lower limit of the 90% CI for CR did not meet the pre-specified threshold of 55%, it should be noted that this historical benchmark was based on the placebo arm of the RATIFY study [22], which assessed the use of midostaurin in combination with chemotherapy for the treatment of *FLT3*^*mut+*^AML in 17 countries globally. Despite being similar in design to the present study, a notable difference to RATIFY was the timing of patient progression from induction to consolidation therapy [22]. In the RATIFY study, bone marrow aspiration was performed on Day 21 of the induction period to determine the need for a second cycle of induction therapy; in addition, bone marrow aspiration was performed within one week after hematologic recovery and no later than Day 60 to assess for responses [22]. Patients with residual AML after a second remission induction were removed from protocol therapy while those with complete response proceeded to consolidation therapy [22]. In contrast, in our study bone marrow assessment could be performed any time after Day 28 per institutional guidelines; additionally, postponing the initiation of consolidation therapy was preferred until full hematologic recovery was achieved, although no threshold was set. Therefore, patients in this study with only CRp/CRi could also proceed to consolidation therapy, which may have reduced the post-induction CR rate. As such, differences in protocol settings and regional practices globally may have impacted the primary endpoint of the CR rate in this interim analysis.

Of the 71 patients with CRc after induction therapy, 55 achieved CR/CRh, indicating that most patients had achieved partial hematologic recovery (defined as neutrophil count ≥500/mm^3^ and platelet count ≥50,000/mm^3^). From the end of the induction to the end of the consolidation period, the number of patients with CR, i.e., with full hematologic recovery, had increased from 41 to 52 at the data cut-off. It is possible that the myelosuppressive effects of *FLT3* inhibitors delayed hematologic recovery in this study [23].

In our study, the CR rate at the end of the induction period (50.0%) was similar to those previously reported in the global phase 3 QuANTUM-First study of patients with *FLT3*-ITD AML receiving either quizartinib plus chemotherapy (54.9% CR) or placebo plus chemotherapy (55.4% CR) [20]. In our *ad hoc* analysis, in which the response definition was modified to more closely align with the definition used in the QuANTUM-First study, CRc rate was 69.5%; this was similar to the CRc rates in the QUANTUM-First study of 71.6% with quizartinib and 64.9% with placebo [20].

Median OS, EFS and RFS could not be estimated in this interim analysis because the data were not mature for these endpoints.

In this interim analysis, approximately half (54.1%) of patients in the phase 2 part achieved MRD negativity, which may contribute to beneficial trends in survival rate. Additionally, the proportion of patients who underwent HSCT was 36.6% at data cut-off, which was similar to the proportion who underwent protocol-specified allogeneic HSCT in the QuANTUM-First study in both the quizartinib (38.1%) and placebo (33.6%) arms after induction and consolidation therapy [20]. Although higher overall transplantation rates were reported previously in the RATIFY study (57.0%) than in our study [22], this is likely because the HSCT data in this study have yet to fully mature. Caution should be taken when making direct comparisons of transplantation rates between this study and the RATIFY or QuANTUM-FIRST studies, due to differences in patient populations and methodology between the trials.

The median time to hematologic recovery after the start date for Cycle 1 of induction therapy in this study (48.0 days) was slower than in a previous phase 3 study of patients with ND AML receiving chemotherapy alone (31–35 days, calculated based on published data) [24]. A similar delay in hematologic recovery (41 days) was reported in a previous phase 1B study of gilteritinib combined with 7+3 cytarabine/idarubicin or cytarabine/daunorubicin induction remission [18], and also in the QuANTUM-First study of quizartinib in combination with chemotherapy versus chemotherapy alone, albeit to a lesser extent (31–36 vs 29 days, respectively) [20]. It appears that hematologic recovery tends to be slower when an FLT3 inhibitor is used in combination with chemotherapy than when chemotherapy alone is evaluated. However, it should be noted that there are differences in the methodology used for measurement of hematologic recovery in each study.

Our findings suggest that gilteritinib has a comparable safety profile to that of other FLT3 inhibitors. Gilteritinib in combination with cytarabine/idarubicin as induction therapy and gilteritinib plus high-dose cytarabine as consolidation therapy was well tolerated in this patient population. In general, the rate of serious AEs observed in the phase 2 part of this interim analysis (47.6%) was similar to that in the quizartinib arm of the QuANTUM-First study (54%) [20]. In addition, the rate of early death (occurring ≤60 days after study drug initiation) was also slightly lower in our study (4.8%) than in the QuANTUM-First study (8%) [20]. No new safety findings were reported in this analysis compared to other FLT3 inhibitors, as published in the QuANTUM-First (quizartinib) or RATIFY (midostaurin) studies, and a similar occurrence of common grade ≥3 AEs was observed across the studies [20, 22].

Our study identified the most appropriate dose of gilteritinib for combination therapy in Asian patients with ND *FLT3*^*mut+*^AML, and provides insights into the efficacy and safety of gilteritinib combination induction and consolidation therapy in this setting. Strengths of our study include the enrollment of patients with either *FLT3*-ITD or *FLT3*-TKD mutations in the phase 2 part, the inclusion of patients over 60 years of age, and the continued inclusion of patients post-HSCT; in contrast, patients over 60 years of age and/or who received HSCT were excluded from the RATIFY study. Additionally, we employed next-generation sequencing analysis to determine MRD after gilteritinib treatment, a method that provides enhanced sensitivity and specificity for the detection of *FLT3* mutations [19].

A key limitation of this study was the differences in protocol settings and regional practices (i.e., initiation of consolidation therapy prior to full hematologic recovery) between Asia and the US/EU. Our study protocol did not require patients to reach full CR at the end of induction therapy, which may have artificially confounded the primary endpoint of this study. Lastly, this study did not include a comparator arm; instead, the increase in CR rate was evaluated using a historical benchmark based on the placebo arm of a previous study (RATIFY) [22].

In conclusion, this interim analysis demonstrated that gilteritinib at a dose of 120 mg/day in combination with induction or consolidation chemotherapy in previously untreated Asian patients with ND *FLT3*^*mut+*^AML, resulted in half of patients achieving CR after induction therapy, while rates of CRc were similar to previous studies. Gilteritinib in combination with chemotherapy was also well tolerated with no new safety findings. Final analyses will be conducted following study completion.

## Supplementary Information

Below is the link to the electronic supplementary material.

Supplementary file1 (PDF 293 KB)

## Data Availability

Researchers may request access to anonymized patient-level data, trial-level data and protocols from Astellas-sponsored clinical trials at https://www.clinicalstudydatarequest.com. For the Astellas criteria on data sharing see: https://clinicalstudydatarequest.com/Study-Sponsors/Study-Sponsors-Astellas.aspx.
