# Supplementary material for: A phase 1/2 study of gilteritinib in combination with chemotherapy in newly diagnosed patients with AML in Asia
Source: Int J Hematol. 2024 Nov 6;121(1):56–67. doi: 10.1007/s12185-024-03840-x (PMC11748473; doi:10.1007/s12185-024-03840-x)
Supplement: Supplementary file 1 — Supplementary file1 (PDF 293 KB) [file 12185_2024_3840_MOESM1_ESM.pdf]

SUPPLEMENTARY INFORMATION

Supplementary Methods

Definition of dose-limiting toxicity

A DLT was defined as any of the following events that occurred during the DLT assessment period, and that was considered to be possibly, probably, or definitely related to induction or consolidation therapies including the study drugs.

1. Any grade 3 or higher non-hematologic or extramedullary toxicity or any events that required dose reduction of gilteritinib. However, the following exceptions were noted:

    - Anorexia, or fatigue
    - Grade 3 nausea and/or vomiting that did not require tube feeding or TPN, and
    - Grade 3 diarrhea that did not require prolonged hospitalization. However, these were limited to the ones that could be managed to grade 2 or lower with standard antiemetic or antidiarrheal medications used at the prescribed dose within 7 days of onset.
    - Grade 3 mucositis that resolved to grade 2 or lower within 7 days of onset
    - Grade 3 pyrexia with neutropenia, with or without infection
    - Grade 3 infection

2. The following hematologic toxicities that occurred after the first dose of gilteritinib and that did not resolve by Day 42 of the last induction therapy cycle or before the

start of the first consolidation cycle, whichever was sooner. However, hematologic toxicity in patients who did not achieve remission was not included in assessment.

- Peripheral neutrophil count <500/mm^3 (grade 4)
- Platelet count <20,000/mm^3 due to bone marrow hypoplasia (except for cases caused by leukemic infiltration or other causes). Bone marrow hypoplasia was defined as bone marrow cellularity less than 20%
- Platelet count <50,000/mm^3 accompanying bleeding (≥ grade 3)
- Platelet count <25,000/mm^3 requiring platelet transfusion (grade 4)

The DLT assessment period for making a decision of whether or not to proceed to the next dose was defined as the shorter of the following 2 periods: 39 days from the start of the treatment with gilteritinib during the induction period, or the period between the start of induction therapy and the start of the first consolidation therapy. For safety assessments during the dose-expansion part, the DLT assessment period included Cycle 1 of consolidation therapy in addition to the period defined above.

Criteria for resuming the study after HSCT

Patients who had a donor identified and achieved a response (CRc or PR) were permitted to undergo HSCT per each institution's assessment and continue to receive the protocol-specified maintenance therapy without leaving the study. However, gilteritinib was stopped and a pre-HSCT visit was performed prior to starting the conditioning regimen for HSCT. Gilteritinib could be resumed after HSCT if the following conditions are met:

- Patient was between 30–90 days post-HSCT

- Patient had successful engraftment as demonstrated by absolute neutrophil count (ANC) ≥500/mm^3 and platelets ≥20000/mm^3 without transfusions
- Patient did not have Grade ≥2 acute graft-versus-host disease
- Patient was in composite complete remission (CRc)

Patients resuming treatment followed procedures from day 1 of the initial or subsequent cycle of maintenance therapy. Patients who did not resume gilteritinib were followed for the survival endpoints.

Determination of MTD and RED

The candidate MTD was the dose at which the posterior mean of DLT incidence was estimated to be closest to 33% when calculated using Bayesian continual reassessment from the status of DLT occurrence accumulated from this study during Cycle 1 of the induction period. Through discussion with the medical expert, investigator, and advisor of medical statistics, the sponsor comprehensively reviewed the data obtained from Cycle 1, and decided the final MTD.

The sponsor decided the RED considering the MTD, safety, pharmacokinetics, and efficacy of gilteritinib. The final RED was decided by the sponsor's responsible person by comprehensively assessing the data obtained from the study and taking into account the discussion held between the sponsor, the medical expert, the investigator, and the advisor of medical statistics.

Protocol-specified laboratory eligibility criteria by study part

Based on the laboratory tests conducted during screening, study patients were required to meet all of the following criteria:

- Phase 1 part
    - Serum aspartate aminotransferase (AST) and alanine aminotransferase (ALT) levels of ≤2.5 × institutional upper limit of normal (ULN)
    - Total serum bilirubin level of ≤1.5 × institutional ULN
    - Serum creatinine level of ≤1.5 × institutional ULN or an estimated glomerular filtration rate (eGFR) of >50 mL/min[†]
- Phase 2 part
    - Serum creatinine ≤1.5 x institutional ULN, or if serum creatinine outside normal range, then glomerular filtration rate (GFR) > 50 mL/min/1.73 m^2 as calculated with the 4-parameter Modification of Diet in Renal Disease (MDRD) equation
    - Serum total bilirubin ≤2.5 mg/dL (43 μmol/L), except for patients with Gilbert's syndrome
    - Serum AST and ALT <3 × ULN.
    - Serum magnesium ≥ institutional lower limit of normal (LLN).
    - Serum potassium ≥ institutional LLN

Definitions of efficacy outcomes

Complete remission (CR) was defined as a morphologically leukemia-free state with full hematologic recovery (defined as neutrophil count ≥1000/mm^3, platelet count ≥100,000/mm^3, bone marrow blasts <5%; no evidence of Auer rods and extramedullary leukemia, and peripheral blood blast count ≤2%.

Complete remission with incomplete platelet recovery (CRp) was defined as a condition that met all of the complete response criteria at the post-baseline visit, except for the unrecovered platelet count (<100,000/mm^3).

Complete remission with partial hematologic recovery (CRh) was defined as a condition with bone marrow blasts <5%, neutrophil count ≥500/mm^3, platelet count ≥50,000/mm^3, no evidence of extramedullary leukemia, and peripheral blood blast count ≤2%, and that also could not be classified as CR.

CR/CRh was defined as achievement of conditions for either CR or CRh, respectively.

CR with incomplete hematologic recovery (CRi) was defined as a condition that met all CR criteria at the post-baseline visit, except for an unrecovered neutrophil count (<1,000/mm^3), and regardless of whether platelet count was recovered. In a further *ad hoc* analysis, CRi data were only counted up to day 60 from day 1 of the last induction cycle; additionally, CRi was defined as a condition that met all of the CR criteria at the post-baseline visit, except for an unrecovered neutrophil count (<1,000/mm^3) but including platelet recovery (≥100,000/mm^3).

Composite CR (CRc) was defined as achievement of one of CR, CRp or CRi.

Partial remission was defined as regeneration of normal hematopoietic cells in the bone marrow, no detectable (or trace of residual) blasts, ≥50% decrease of blasts in the bone-marrow aspirate and total bone marrow blasts of 5–25%. There should be no evidence of extramedullary leukemia.

Relapse was defined as reappearance after CR, CRp or CRi of leukemic blasts in the peripheral blood (>2%) or ≥5% blasts in the bone marrow aspirate not attributable to any other cause, or reappearance or new appearance of extramedullary leukemia.

Minimal residual disease (MRD) negativity was defined as a summed *FLT3*-ITD signal ratio ≤10^{-4} for any post-baseline sample. MRD was determined using a polymerase chain reaction (PCR) amplified next-generation sequencing method that provides the sensitivity to detect ≥1 *FLT3*-ITD-containing leukemic cell among 10,000 cells containing the wild-type allele [19].

Overall survival (OS) was defined as the time from the date of first dose on Day 1 to the date of death due to any cause. Patients still alive or lost to follow-up were censored at the time they were last known to be alive.

Event-free survival (EFS) was defined as the time from the date of first dose of study regimen (Day 1) until the date of documented relapse, treatment failure, or death from any cause, whichever occurred first. For patients with none of these events, EFS was censored at the date of last disease assessment.

Relapse-free survival (RFS) was defined as the time from the date of achievement of first CRc until relapse or death from any cause, whichever came first. For a patient who was not known to have relapsed or died, RFS was censored on the date of last relapse-free disease assessment date.

Time to hematologic recovery after a treatment cycle was defined as the time from the start date of the treatment for each treatment cycle until the date of neutrophil count ≥1,000/mm^3 and platelet count ≥100,000/mm^3. Patients with none of these events were censored at the date of last assessment.

SUPPLEMENTARY FIGURES

Figure S1: Patient disposition by study part

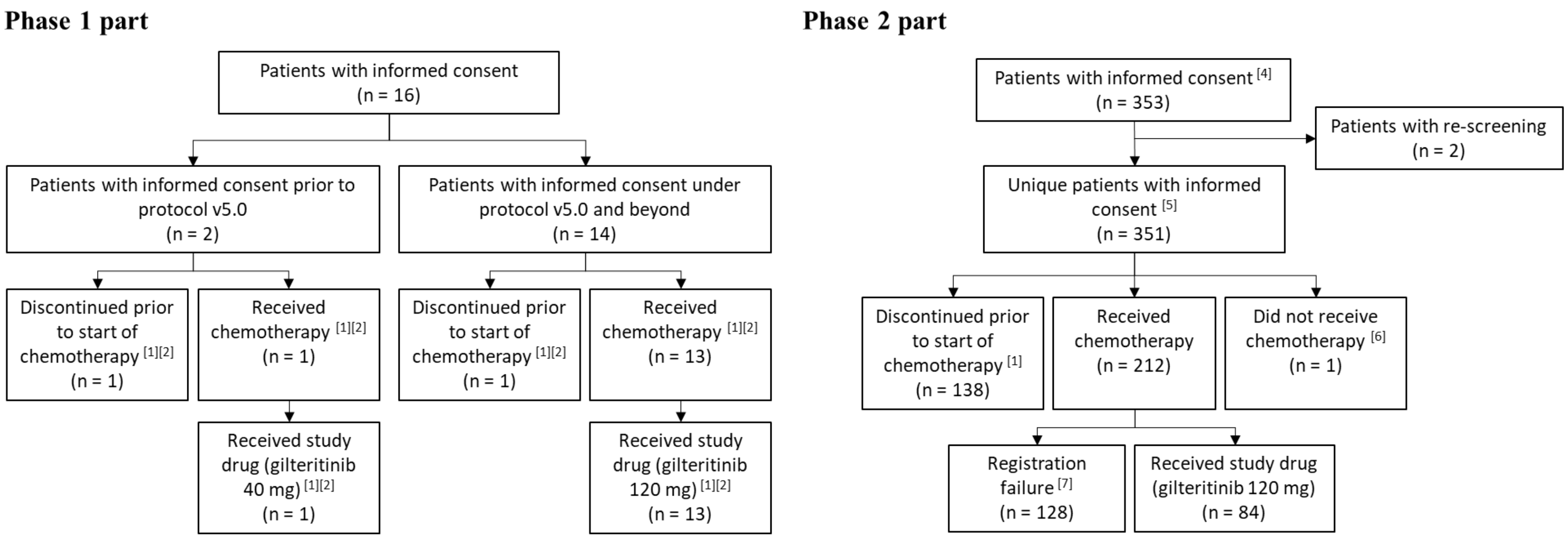

[1] Patients who signed informed consent but discontinued before start of treatment (chemotherapy) were screen failures.

[2] Patients who signed informed consent prior to protocol version 5.0 (in which all patients received a starting gilteritinib dose of 120 mg/day).

[3] Patients who signed informed consent under protocol version 5.0 (in which all patients received a starting gilteritinib dose of 120 mg/day) or beyond.

[4] Patients who were screened multiple times were counted for each screening attempt.

[5] Patients who were screened multiple times were only counted once.

[6] This patient was pre-registered but did not receive chemotherapy.

[7] Patients who received chemotherapy but discontinued before start of gilteritinib were considered as registration failures.

One patient who received gilteritinib 40 mg in the phase 1 part was excluded from all analyses.

SUPPLEMENTARY TABLES

Table S1: Patients by analysis population and study part

Analysis set	Dose-evaluation (N = 3)	Dose-expansion (N = 10)	Total (N = 13)
**Phase 1 part**			
Patients who received chemotherapy	3 (100.0)	10 (100.0)	13 (100.0)
FAS [1]	3 (100.0)	9 (90.0)	12 (92.3)
SAF [2]	3 (100.0)	10 (100.0)	13 (100.0)
DDAS [3]	3 (100.0)	10 (100.0)	13 (100.0)
PKAS [4]	3 (100.0)	10 (100.0)	13 (100.0)
PDAS [5]	0	0	0
**Phase 2 part**			
Patients who received chemotherapy	-	-	212 (100.0)
Patients who registered in the study	-	-	84 (39.6)

FAS [6]	-	-	82 (38.7)
SAF [2]	-	-	84 (39.6)
MAS [7]	-	-	61 (28.8)
TTE-FAS [8]	-	-	84 (39.6)
PKAS [9]	-	-	83 (39.2)

Data are presented as n (%).

[1] All patients who received at least 1 dose of gilteritinib and were assessed for at least 1 efficacy variable after gilteritinib administration.

[2] All patients who received at least 1 dose of gilteritinib.

[3] Included patients who did not fall under either of the following criteria: received less than 80% of the intended dose of gilteritinib during DLT assessment period specified in each part; unable to assess safety adequately during DLT assessment period specified in each part.

[4] Patients who received gilteritinib, from whom samples for drug concentration measurement were collected and obtained at least once after gilteritinib administration.

[5] Patients who received gilteritinib, from whom samples for pharmacodynamic assessment were collected and obtained at least once after gilteritinib administration.

[6] All patients who received at least 1 dose of gilteritinib and had at least 1 post-baseline bone marrow assessment.

[7] Patients who were registered in the study and received at least one dose of gilteritinib, were centrally confirmed as *FLT3*-ITD positive at screening, and had a baseline and at least one post-baseline sample with MRD data.

[8] All patients who received at least one dose of gilteritinib with or without post-baseline bone marrow assessments.

[9] Patients who received gilteritinib, from whom samples for drug concentration measurement were collected for at least one time point after gilteritinib administration, and from whom drug concentration measurement had been obtained.

One patient who received gilteritinib 40 mg in the phase 1 part was excluded from all analyses.

DDAS, dose-determining analysis set; DLT, dose-limiting toxicity; FAS, full analysis set; FLT3, FMS-like tyrosine kinase 3; ITD, internal tandem duplication; MAS, MRD analysis set; MRD, minimal residual disease; PKAS, pharmacokinetic analysis set; PDAS, pharmacodynamic analysis set; SAF, safety analysis set; TTE-FAS, time-to-event full analysis set.

Table S2: AML disease history (SAF)

	Phase 1 part			Phase 2 part
**Parameter**	**Dose-evaluation (N = 3)**	**Dose-expansion (N = 10)**	**Total (N = 13)**	**Total (N = 84)**
Duration of disease (months)				
Mean (SD)	0.09 (0.04)	0.17 (0.14)	0.15 (0.13)	0.17 (0.10)
Median (min–max)	0.07 (0.1–0.1)	0.13 (0.0–0.5)	0.13 (0.0–0.5)	0.16 (0.0–0.5)
FAB classification subtype				
M0: minimally differentiated acute myeloblastic leukemia	0	1 (10.0%)	1 (7.7%)	3 (3.8%)
M1: acute myeloblastic leukemia, without maturation	1 (33.3%)	2 (20.0%)	3 (23.1%)	24 (30.4%)

M2: AML with differentiation	2 (66.7%)	3 (30.0%)	5 (38.5%)	31 (39.2%)
M3: acute promyelocytic leukemia	0	0	0	0
M4: acute myelomonocytic leukemia	0	4 (40.0%)	4 (30.8%)	14 (17.7%)
M5: acute monoblastic leukemia	0	0	0	7 (8.9%)
M6: acute erythroid leukemia	0	0	0	0
M7: acute megakaryocytic leukemia	0	0	0	0
Missing	-	-	-	5 (6.0%)
Risk status with specific cytogenetic patterns				

Favorable: inv(16)	0	1 (10.0%)	1 (7.7%)	5 (6.0%)
Favorable: t(16;16)	0	0	0	0
Favorable: t(8;21)	0	1 (10.0%)	1 (7.7%)	4 (4.8%)
Favorable: t(15;17)	0	0	0	0
Intermediate: normal	2 (66.7%)	3 (30.0%)	5 (38.5%)	46 (54.8%)
Intermediate: +8	0	0	0	4 (4.8%)
Intermediate: +6	0	0	0	0
Intermediate: -y	0	1 (10.0%)	1 (7.7%)	0
Unfavorable: del5q	0	0	0	0
Unfavorable: -5	0	0	0	0

Unfavorable: del7q	0	0	0	0
Unfavorable: -7	0	0	0	1 (1.2%)
Unfavorable: complex	1 (33.3%)	1 (10.0%)	2 (15.4%)	4 (4.8%)
Unknown	0	3 (30.0%)	3 (23.1%)	7 (8.3%)
Other	0	0	0	13 (15.5%)

AML, acute myeloid leukemia; del, deletion; FAB, French-American-British; *FLT3*, FMS-like tyrosine kinase 3; ITD, internal tandem duplication; inv, inversion; p, short arm of chromosome; q, long arm of chromosome; TKD, tyrosine kinase domain; t, translocation; v, variable chromosome.

Table S3: Grade 3 or higher TEAEs (SAF)

	Phase 1 part (N=13)	Phase 2 part (N=84)	Combined phase 1 & 2 parts (N=97)
Overall, n (%)	13 (100.0)	79 (94.0)	92 (94.8)
Blood and lymphatic system disorders, n (%)	13 (100.0)	58 (69.0)	71 (73.2)
Febrile neutropenia	12 (92.3)	54 (64.3)	66 (68.0)
Anemia	8 (61.5)	17 (20.2)	25 (25.8)
Thrombocytopenia	4 (30.8)	13 (15.5)	17 (17.5)
Neutropenia	5 (38.5)	9 (10.7)	14 (14.4)
Leukopenia	4 (30.8)	7 (8.3)	11 (11.3)

Bone marrow failure	0	4 (4.8)	4 (4.1)
Cardiac disorders, n (%)	0	6 (7.1)	6 (6.2)
Cardiac failure	0	3 (3.6)	3 (3.1)
Myocardial infarction	0	1 (1.2)	1 (1.0)
Tachycardia	0	1 (1.2)	1 (1.0)
Stress cardiomyopathy	0	1 (1.2)	1 (1.0)
Gastrointestinal disorders, n (%)	3 (23.1)	7 (8.3)	10 (10.3)
Diarrhea	3 (23.1)	2 (2.4)	5 (5.2)
Abdominal pain	0	1 (1.2)	1 (1.0)
Colitis	0	1 (1.2)	1 (1.0)

Duodenal ulcer	0	1 (1.2)	1 (1.0)
Enterocolitis	0	1 (1.2)	1 (1.0)
Ileus	0	1 (1.2)	1 (1.0)
Nausea	0	1 (1.2)	1 (1.0)
Stomatitis	0	1 (1.2)	1 (1.0)
General disorders and administration site conditions, n (%)	1 (7.7)	5 (6.0)	6 (6.2)
Pyrexia	1 (7.7)	5 (6.0)	6 (6.2)
Hepatobiliary disorders, n (%)	1 (7.7)	6 (7.1)	7 (7.2)
Hepatic function abnormal	1 (7.7)	4 (4.8)	5 (5.2)
Drug-induced liver injury	0	2 (2.4)	2 (2.1)

Cholecystitis	0	1 (1.2)	1 (1.0)
Immune system disorders, n (%)	0	2 (2.4)	2 (2.1)
Graft-versus-host disease	0	1 (1.2)	1 (1.0)
Acute graft-versus-host disease	0	1 (1.2)	1 (1.0)
Infections and infestations, n (%)	5 (38.5)	35 (41.7)	40 (41.2)
Pneumonia	2 (15.4)	9 (10.7)	11 (11.3)
Bacteraemia	1 (7.7)	8 (9.5)	9 (9.3)
Sepsis	2 (15.4)	6 (7.1)	8 (8.2)
Device related infection	2 (15.4)	3 (3.6)	5 (5.2)
Septic shock	0	3 (3.6)	3 (3.1)

Bronchopulmonary aspergillosis	0	2 (2.4)	2 (2.1)
Appendicitis	0	1 (1.2)	1 (1.0)
Brain abscess	0	1 (1.2)	1 (1.0)
Cellulitis	0	1 (1.2)	1 (1.0)
Clostridium difficile colitis	0	1 (1.2)	1 (1.0)
Liver abscess	0	1 (1.2)	1 (1.0)
Meningitis	0	1 (1.2)	1 (1.0)
Pulmonary mycosis	0	1 (1.2)	1 (1.0)
Sinusitis	0	1 (1.2)	1 (1.0)
Splenic abscess	0	1 (1.2)	1 (1.0)

Ureteritis	0	1 (1.2)	1 (1.0)
Abdominal abscess	0	1 (1.2)	1 (1.0)
Pneumonia fungal	0	1 (1.2)	1 (1.0)
Lymphadenitis bacterial	0	1 (1.2)	1 (1.0)
Root canal infection	0	1 (1.2)	1 (1.0)
Injury, poisoning and procedural complications, n (%)	0	3 (3.6)	3 (3.1)
Allergic transfusion reaction	0	2 (2.4)	2 (2.1)
Spinal compression fracture	0	1 (1.2)	1 (1.0)
Investigations, n (%)	11 (84.6)	47 (56.0)	58 (59.8)
Platelet count decreased	7 (53.8)	27 (32.1)	34 (35.1)

Neutrophil count decreased	6 (46.2)	23 (27.4)	29 (29.9)
White blood cell count decreased	8 (61.5)	13 (15.5)	21 (21.6)
Alanine aminotransferase increased	0	13 (15.5)	13 (13.4)
Aspartate aminotransferase increased	0	9 (10.7)	9 (9.3)
Lymphocyte count decreased	1 (7.7)	5 (6.0)	6 (6.2)
Gamma-glutamyltransferase increased	0	5 (6.0)	5 (5.2)
Liver function test abnormal	3 (23.1)	0	3 (3.1)
Electrocardiogram QT prolonged	0	2 (2.4)	2 (2.1)
Amylase increased	0	1 (1.2)	1 (1.0)
Blood bilirubin increased	0	1 (1.2)	1 (1.0)

Lipase increased	0	1 (1.2)	1 (1.0)
Ejection fraction decreased	0	1 (1.2)	1 (1.0)
Metabolism and nutrition disorders, n (%)	2 (15.4)	19 (22.6)	21 (21.6)
Hypokalaemia	0	12 (14.3)	12 (12.4)
Hyponatraemia	0	5 (6.0)	5 (5.2)
Decreased appetite	2 (15.4)	1 (1.2)	3 (3.1)
Hypocalcaemia	0	2 (2.4)	2 (2.1)
Hypophosphataemia	0	2 (2.4)	2 (2.1)
Hypermagnesaemia	0	1 (1.2)	1 (1.0)
Tumour lysis syndrome	1 (7.7)	0	1 (1.0)

Steroid diabetes	0	1 (1.2)	1 (1.0)
Neoplasms benign, malignant and unspecified (incl. cysts and polyps), n (%)	0	1 (1.2)	1 (1.0)
Neuroendocrine tumor	0	1 (1.2)	1 (1.0)
Nervous system disorders	0	5 (6.0)	5 (5.2)
Syncope	0	2 (2.4)	2 (2.1)
Brain stem infarction	0	1 (1.2)	1 (1.0)
Cerebral infarction	0	1 (1.2)	1 (1.0)
Presyncope	0	1 (1.2)	1 (1.0)
Renal and urinary disorders, n (%)	0	2 (2.4)	2 (2.1)
Tubulointerstitial nephritis	0	1 (1.2)	1 (1.0)

Acute kidney injury	0	1 (1.2)	1 (1.0)
Respiratory, thoracic and mediastinal disorders, n (%)	0	4 (4.8)	4 (4.1)
Dyspnea	0	1 (1.2)	1 (1.0)
Hemothorax	0	1 (1.2)	1 (1.0)
Hiccups	0	1 (1.2)	1 (1.0)
Pulmonary hemorrhage	0	1 (1.2)	1 (1.0)
Skin and subcutaneous tissue disorders, n (%)	0	8 (9.5)	8 (8.2)
Rash maculo-papular	0	4 (4.8)	4 (4.1)
Acute febrile neutrophilic dermatosis	0	1 (1.2)	1 (1.0)

Drug eruption	0	1 (1.2)	1 (1.0)
Rash	0	1 (1.2)	1 (1.0)
Urticaria	0	1 (1.2)	1 (1.0)
Vascular disorders, n (%)	0	2 (2.4)	2 (2.1)
Hypertension	0	2 (2.4)	2 (2.1)

Data are presented as system organ class and preferred term, based on MedDRA v23.0.

SAF, safety analysis set; TEAE, treatment-emergent adverse event.

Table S4. Derived response rates using modified CRi definition (*ad hoc* analysis) (FAS)

	Phase 2 patients (N=82)	
**BOR**	**After induction therapy, n (%) [95% CI]**	**By 60 days after day 1 of the last induction cycle, n (%) [95% CI]**
CR	41 (50.0) [38.7, 61.3]	34 (41.5) [30.7, 52.9]
CRp	11 (13.4) [6.9, 22.7]	11 (13.4) [6.9, 22.7]
CRi	12 (14.6) [7.8, 24.2] [1]	12 (14.6) [7.8, 24.2] [2]
CRc	64 (78.0) [67.5, 86.4]	57 (69.5) [58.4, 79.2]
CR without MRD	15 (18.3) [10.6, 28.4]	14 (17.1) [9.7, 27.0]
CRc without MRD	19 (23.2) [14.6, 33.8]	18 (22.0) [13.6, 32.5]

Exact 95% CI were estimated using the binomial distribution. For subjects who undergo HSCT, any response assessment data after HSCT are not included in this table.

[1] For this *ad hoc* analysis, CRi was defined as a condition that met all of the CR criteria at the post-baseline visit, except for an unrecovered neutrophil count (<1,000/mm^3) but including platelet recovery (≥100,000/mm^3).

[2] For this *ad hoc* analysis, CRi data were only counted up to day 60 from day 1 of the last induction cycle, and CRi was defined as a condition that met all of the CR criteria at the post-baseline visit, except for an unrecovered neutrophil count (<1,000/mm^3) but including platelet recovery (≥100,000/mm^3).

BOR, best overall response (patients who achieved CR in induction therapy were counted as CR even if not reported with CR in consolidation and maintenance therapy); CI, confidence interval; CR, complete remission; CRc, composite complete remission (CR + CRp + CRi); CRi, complete remission with incomplete hematological recovery; CRp, complete remission with incomplete platelet recovery; FAS, full analysis set; MRD minimal residual disease.

Table S5: Summary of overall survival and event-free survival events (TTE-FAS)

	**Total (N=84)**
**OS**	
OS rate, % (95% CI) [1]	
Month 6	91.8 (82.4, 96.3)
Month 12	86.6 (73.9, 93.4)
Death, n (%)	8 (9.5)
Censored, n (%)	76 (90.5)
**EFS**	
EFS events, n (%) [2]	17 (20.2)
Relapse	1 (1.2)
Treatment failure	10 (11.9)
Death	6 (7.1)
Censored, n (%)	67 (79.8)

**RFS** [3]	n=75
RFS events, n (%) [4]	12 (16.0)
Relapse	9 (12.0)
Death	3 (4.0)
Censored, n (%)	63 (84.0)

CI: confidence interval; OS, overall survival; RFS, relapse-free survival; TTE-FAS, time-to-event full analysis set.

[1] Survival rate and 95% CI were estimated using Kaplan-Meier method and Greenwood formula.

[2] Patients were summarized under the event categories that occurred first. If treatment failure and death occurred on the same day, patients were summarized under death.

[3] RFS was only applicable to patients with a best overall response (across induction, consolidation, and maintenance therapies) of CRc.

[4] Patients were summarized under the event categories that occurred first.

Table S6: Potentially clinically significant values in laboratory tests (SAF)

	Phase 1 part (N=13)	Phase 2 part (N=84)
ALT, n/N (%)		
>3 x ULN	5/13 (38.5)	23/83 (27.7)
>5 x ULN	2/13 (15.4)	9/83 (10.8)
>8 x ULN	2/13 (15.4)	6/83 (7.2)
>10 x ULN	2/13 (15.4)	2/83 (2.4)
>20 x ULN	0	0
AST, n/N (%)		
>3 x ULN	4/13 (30.8)	12/83 (14.5)
>5 x ULN	2/13 (15.4)	4/83 (4.8)
>8 x ULN	1/13 (7.7)	1/83 (1.2)
>10 x ULN	1/13 (7.7)	0
>20 x ULN	0	0

ALT or AST, n/N (%)		
>3 x ULN	5/13 (38.5)	25/84 (29.8)
>5 x ULN	3/13 (23.1)	10/84 (11.9)
>8 x ULN	3/13 (23.1)	6/84 (7.1)
>10 x ULN	3/13 (23.1)	2/84 (2.4)
>20 x ULN	0	0
Total bilirubin, n/N (%)		
>2 x ULN	0	6/83 (7.2)
ALP, n/N (%)		
>1.5 x ULN	2/13 (15.4)	36/83 (43.4)
ALT and/or AST >3 x ULN AND total bilirubin >2 x ULN, n/N (%) [1]	0	2/84 (2.4)
ALT and/or AST >3 x ULN AND ALP <2 x ULN AND total bilirubin >2 x ULN, n/N (%) [1]	0	0

[1] Combination of values measured within same sample.

ALT, alanine aminotransferase; ULN, upper limit of normal; AST, aspartate aminotransferase; ALP, alkaline phosphatase.

Table S7: Potentially clinically significant vital signs (SAF)

**Vital sign criteria** [1]	**Phase 1 part (N=13)**	**Phase 2 part (N=84)**
Systolic blood pressure (mmHg):		
≥180 mmHg and ≥20 mmHg change from baseline	-	1/84 (1.2)
Diastolic blood pressure (mmHg):		
≥105 mmHg and ≥15 mmHg change from baseline	-	1/84 (1.2)
Pulse (bpm):		
≥120 bpm and ≥15 bpm change from baseline	-	4/84 (4.8)

The denominator was the number of patients who had at least 1 non-missing value during treatment.

[1] For each patient, the worst change from baseline from all post-baseline measurements was used.

Table S8: QTcF values (SAF)

Mean value (msec)	Phase 1 part, N=13	Phase 2 part, N=84
**Baseline**	n=8	n=84
≤450	8 (100)	83 (98.8)
>450–≤480	0	1 (1.2)
>480–≤500	0	0
>500	0	0
**Maximum post-baseline value**	n=8	n=84
≤450	6 (75.0)	65 (77.4)
>450–≤480	2 (25.0)	15 (17.9)
>480–≤500	0	3 (3.6)
>500	0	1 (1.2)
**Maximum post-baseline change**	n=8	n=84

<0	0	0
≥0–≤30	3 (37.5)	45 (53.6)
>30–≤60	4 (50.0)	31 (36.9)
>60	1 (12.5)	8 (9.5)

QTcF, QT interval corrected for heart rate using Fridericia's factor; SAF, safety analysis set.

For each timepoint, the mean value of observed QTc was used for each patient. Percentages were calculated as the total number of patients within the maximum value category divided by the total number of patients with a non-missing value. For each time point, the mean value of observed QTc result was used for each patient.

'n' corresponds to the number of patients with a non-missing value.

Table S9: Gilteritinib trough plasma concentrations in the induction and consolidation periods of the phase 2 part (PKAS)

	Induction period		Consolidation period	
	Cycle 1		Cycle 1	
	Day 15	Day 21	Day 8	Day 15
n	83	80	53	54
Mean (SD), ng/mL	522 (331)	647 (518)	244 (154)	373 (253)
% CV	63.4	80.1	63.2	67.9
Median (min–max), ng/mL	431 (122–2020)	527 (13.7–2810)	216 (32.8–740)	278 (68.5–1450)
Geometric mean, ng/mL	437	469	203	303
Geometric % CV	66.1	113.7	69.3	74.2

CV, coefficient of variation; PKAS, pharmacokinetic analysis set; SD, standard deviation
